# EVI1 promotes epithelial-to-mesenchymal transition, cancer stem cell features and chemo−/radioresistance in nasopharyngeal carcinoma

**DOI:** 10.1186/s13046-019-1077-3

**Published:** 2019-02-15

**Authors:** Yaoyong Lu, Yingying Liang, Xin Zheng, Xubin Deng, Wendong Huang, Gong Zhang

**Affiliations:** 1 Department of Oncology (Section 3), Gaozhou People’s Hospital, Gaozhou, Guangdong China; 2 Affiliated Cancer Hospital & Institute of Guangzhou Medical University, Guangzhou, China; 3 Yanling Hospital of Southern Medical University, Guangzhou, China; 4 Department of Pharmacy, Maoming People’s Hospital, Maoming, Guangdong China; 5 Department of Radiotherapy, People’s Hospital of Shanxi Province, Taiyuan, China

**Keywords:** EVI1, Epithelial mesenchymal transition, Cancer stem cells, Nasopharyngeal carcinoma

## Abstract

**Background:**

Aberrant EVI1 expression is frequently reported in cancer studies; however, its role in nasopharyngeal carcinoma (NPC) has not been examined in detail. The aim of the present study is to investigate the involvement of EVI1 in progression and prognosis of NPC.

**Methods:**

RT-PCR, immunohistochemistry and western blot assays were used to examine the expression of EVI1 in NPC tissues and cell lines. Fluorescence in situ hybridization assay was used to examine the amplification of EVI1 in NPC tissues. The biological effect of EVI1 was determined by both in vitro and in vivo studies. The dual-luciferase reporter assay was performed to confirm that EVI1 bind at E-cadherin andβ-catenin promoters. The ChIP, EMSA, and coimmunoprecipitation combined with mass spectrometry assays were used to analyze the EVI1 regulated proteins.

**Results:**

EVI1 expression level was up-regulated in NPC tissues and cell lines. EVI1 was amplificated in NPC tissues. We observed that EVI1 down-regulation decreased the cell proliferation and invasive capacity of NPC cells in vitro and in vivo. EVI1, snail, and HDAC1 formed a co-repressor complex to repress E-cadherin expression and ultimately contributed to epithelial mesenchymal transition (EMT) phenotype in NPC cells. In another way, EVI1 directly bound at β-catenin promoter and activated its expression. β-catenin mediated EVI1’s function on cancer stem cells (CSCs) properties. EVI1 up-regulation predicted unfavorable prognosis and contributed to chemo/radio-resistance in NPC cells. Finally, we constructed arsenic trioxide-loaded nanoparticles (ALNPs) and revealed that ALNPs exerted anti-tumor effect in NPC cells.

**Conclusions:**

Our data indicated that EVI1 played an oncogenic role in NPC growth and metastasis and that EVI1 might serve as a novel molecular target for the treatment of NPC.

**Electronic supplementary material:**

The online version of this article (10.1186/s13046-019-1077-3) contains supplementary material, which is available to authorized users.

## Background

Nasopharyngeal carcinoma (NPC), which arises from nasopharyngeal epithelial cells, is highly prevalent in East Asia, especially in southern China. Epstein-Barr virus (EBV) exposure, diet and genetic factors together contribute to NPC initiation. NPC has the potential to metastasize to cervical lymph nodes or distant organs [1]. Many NPC patients are already in the late stage at diagnosis [2]. Therefore, it is urgent to clarify the molecular pathogenesis underlying NPC.

EVI1 is a zinc finger transcription regulator encoded in human chromosome 3q26 that undergoes frequent rearrangements that can activate EVI1 expression [3]. Overexpression of EVI1 plays a critical role in many aggressive forms of cancer, including myeloid leukemia, colon cancer, breast cancer, pancreatic cancer and ovarian cancer [4–6]. However, the biological role and underlying mechanism of EVI1 in NPC are seldom reported.

The epithelial-mesenchymal transition (EMT) was originally described in the context of normal cell differentiation during early development; recently, it was well documented that EMT contributes to cancer metastasis [7]. Loss of E-cadherin is required for EMT to occur [8]. Several transcription factors (snail, Zeb, and Twist), which contribute to epigenetic silencing at the E-cadherin promoter in the form of hypermethylation or histone deacetylation, are known as E-cadherin repressors [9, 10].

Interestingly, studies have well demonstrated that EMT plays a significant role not only in tumor metastasis but also in tumor recurrence, which is highly linked to the biology of cancer stem cells (CSCs) [11, 12]. A series of proteins (e.g., CD44, CD133, and ALDH1) are widely used as CSC markers [13]. Our previous studies have identified ALDH1 and PKH26 as functional markers for identifying CSCs in NPC [14, 15]. It is believed that CSCs contribute to tumor recurrence, relapse and resistance to chemo−/radiotreatment [16–18]. Chemotherapy combined with radiotherapy is the standard treatment for NPC [19]. Our previous studies have demonstrated that targeting CSCs in NPC overcomes radioresistance [15].

In the current study, we explored the biological role of EVI1 in NPC. We found that the EVI1 expression level was elevated in NPC tissues and cell lines. EVI1 interacted with snail and HDAC1 to corepress E-cadherin expression and thereby induce EMT. In addition, EVI1 promoted CSC properties by elevating CSC markers (SOX2 and c-myc). EVI1 activated the Wnt/β-catenin pathway, which is involved in promoting CSC properties. Our study suggests that EVI1 is a therapeutic target for NPC.

## Materials and methods

### Cell culture and samples collection

NPC cell lines were maintained in our lab. These cells were cultured in RPMI 1640 medium (Invitrogen) with 10% FBS. The immortalized nasopharyngeal epithelial cell line NP-69 cells were cultured in were cultured in keratinocyte serum-free medium (KSFM), supplemented with human recombinant epidermal growth factor (rEGF) and bovine pituitary extract. 293 T cells were cultured in Dulbecco’s modified Eagle’s medium, supplemented with 10% fetal bovine serum.

NPC samples were collected from Cancer Center of Guangzhou Medical University and Gaozhou People’s Hospital. This study was conducted with the approval of the Ethics Committees of these hospitals.

### RT-PCR assay

The total RNA was extracted from NPC cells and tissues by using Trizol, followed by transcribed into cDNA. The primers were listed in Table 1.

**Table 1:** Primers used in the study

	Forward	Reverse
E-cadherin	CGAGAGCTACACGTTCACGG	GGGTGTCGAGGGAAAAATAGG
E-cadherin (promoter)	ACTCCAGGCTAGAGGGTCAC	CCGCAAGCTCACAGGTGCTTTGCAGTTCC
EVI1	TTGAGGCCCGTTTAGATACCA	GCTTTCCTTGGAGCAATGTAGTT
GAPDH	GATCATCAGCAATGCCTCC	TCCACGATACCAAAGTTGTC
HDAC1	TAACTATCAAAGGACACGCC	TACGAATGGTGTAACCACC

### Western blot assay, immunofluorescence assay and immunohistochemistry

To perform western blot assay, total proteins were extracted from NPC cells and were subsequently resolved, transferred to polyvinylidene fluoride (PVDF) membranes. After blocked in 5% non-fat milk/TBST, the membranes were incubated with primary antibodies and second antibodies, respectively. Then the images were captured with ChemiDocTM CRS+ Molecular Imager.

The immunofluorescence assay was carried out as our previous study described [20].

The immunohistochemistry assay was performed as previously described [21].

### Oligonucleotide transfection and lentivirus infection

siRNAs for EVI1 and β-catenin were synthesized by GeneChem(Shanghai, China). The si-RNAs sequences were previously described [22, 23]. PcDNA.3.1-β-catenin and PcDNA.3.1-EVI1 were purchased from Santa Cruz. Oligonucleotide transfection was performed with Lipofectamine 2000 reagent (Invitrogen).

Lentiviral particles carrying EVI1(LV-EVI1), short hairpin RNA targeting EVI1 (sh-EVI1) and their control(LV-ctrl and sh-ctrl) were designed and synthesized GeneChem(Shanghai, China). Cells were infected with viral particles in the presence of 8 μg/ml polybrene.

### MTT and colony formation assay

Cell viability was determined by the MTT assay. Briefly, 1000 cells/well were seeded in 96-well plates and allowed to attach overnight. Medium was removed and cells were washed with PBS. MTT was added to each well and the cells were incubated at 37 °C for 2 h. The reaction was stopped by lysing the cells with dimethyl sulfoxide (DMSO). The optical density was obtained at a wavelength of 570 nm using spectrophotometric analysis and the percentage cell viability was calculated.

Cell survival rate was determined by the colony assay. Briefly, 200 cells/well were seeded in 6-well plates and allowed to grow for two weeks. Colonies were fixed with paraformaldehyde, stained with giemsa and counted using a microscope.

### Matrigel invasion assay

The matrigel invasion assay was performed as previously described [24].

### Side population, tumor sphere formation assays

The side population and tumor sphere formation assays were carried out as our previous study described [20].

### ALDH1 and PKH26 sorting

The ALDH1 and PKH26 analysis were previously described, respectively [14, 15].

### Luciferase reporter assays

For E-cadherin promoter assay, cells in 24-well plate were co-transfected with E-cadherin promoter or/and pcDNA.3.1-EVI1. 36 h after transfection, dual luciferase reporter assay was carried out by using a Dual-Luciferase Assay Kit (Promega).

The TOP/FOP Flash reporter assay was carried out according to our previous study [20].

### Chromatin immunoprecipitation

We identified the EVI1- binding sites on β-catenin promoter region by using JASPAR (http://jaspar.genereg.net) and ALGGEN (http://alggen.lsi.upc.es) database.

We immunoprecipitated DNA-protein complexes from NPC cells by sing the Chromatin Immunoprecipitation Kit (Thermo Scientific, USA). IgG was used as a control. RT-PCR assay was used to determine the enrichment of β-catenin promoter region.

### Coimmunoprecipitation (co-IP)

The cells were resuspended in RIPA lysis buffer, followed by immunoprecipitated with anti-EVI1, anti-HDAC1 or anti-Snail antibodies, respectively. After incubated overnight at 4 °C, the proteins were further incubated with protein G agarose beads. Finally, the immunocomplexes were subjected to coomassie brilliant blue staining, mass spectrometry and western blot analysis. Anti-IgG was used as a negative control.

### Human serum albumin loaded arsenic trioxide preparation

The human serum albumin loaded arsenic trioxide was prepared as previously described [25, 26].

### Cytotoxicity assay

MTT assay was performed to determine 5-Fu, ALNP and radiation sensitivity. For 5-Fu and ALNP toxicity assay, cells (5 × 10^3^/well) were seeded in 96-well plates and treated with different concentrations of 5-Fu or ALNP. For radiation sensitivity assay, cells (5 × 10^3^/well) were seeded in 96-well plates and treated with different Dose of radiation. Calculated rates were used for curve fitting and half maximal inhibitory concentration (IC50) calculations.

### Xenograft experiments

Animal studies were conducted with the principles and procedures approved by the Committee on the Ethics of Animal Experiments of Guangzhou Medical University**.** To analyze EVI1’s effect on cell growth, sh-ctrl or sh-EVI1 cells were mixed with growth factor-reduced phenol red-free Matrigel and were subcutaneously injected into both flanks of nude mice. Four weeks after implantation, xenografts were removed from the mice and weighed. Tumor volume was calculated using the following formula: 4π/3 × (width/2)2 × (length/2).

Invasion assays were performed as previously described [27].

To analyze the effect of ionizing radiation and si-EVI1 as combination therapy, NPC cells were subcutaneously injected into athymic nude mice. When the tumors reached a size of 70 mm^3^, mice were randomly distributed to 4 groups(ctrl, ALNPs, radiation, ALNPs+radiation). PBS and ALNPs (2 mg/kg/per mouse) were intraperitoneally injected into the mice every four days for eight times. Mice received 12 Gy dosage of radiation at day 7 and 14, respectively, with an X-ray generator.

To analyze the effect of 5-Fu and si-EVI1 as combination therapy, NPC cells were subcutaneously injected into athymic nude mice. When the tumors reached a size of 70 mm^3^, mice were randomly distributed to 4 groups(ctrl, ALNPs, 5-Fu, ALNPs+ 5-Fu). PBS, ALNPs (2 mg/kg/per mouse) and 5-Fu (20 mg/kg/per mouse) were intraperitoneally injected into the mice every four days for eight times.

### Statistical analysis

Numerical data are expressed as the mean ± standard deviation (SD). The difference between means was analyzed using Student’s t-test and differences with *p* < 0.05 were considered statistically significant.

## Results

### EVI1 expression was elevated in NPC tissues and cell lines

**Fig. 1:**
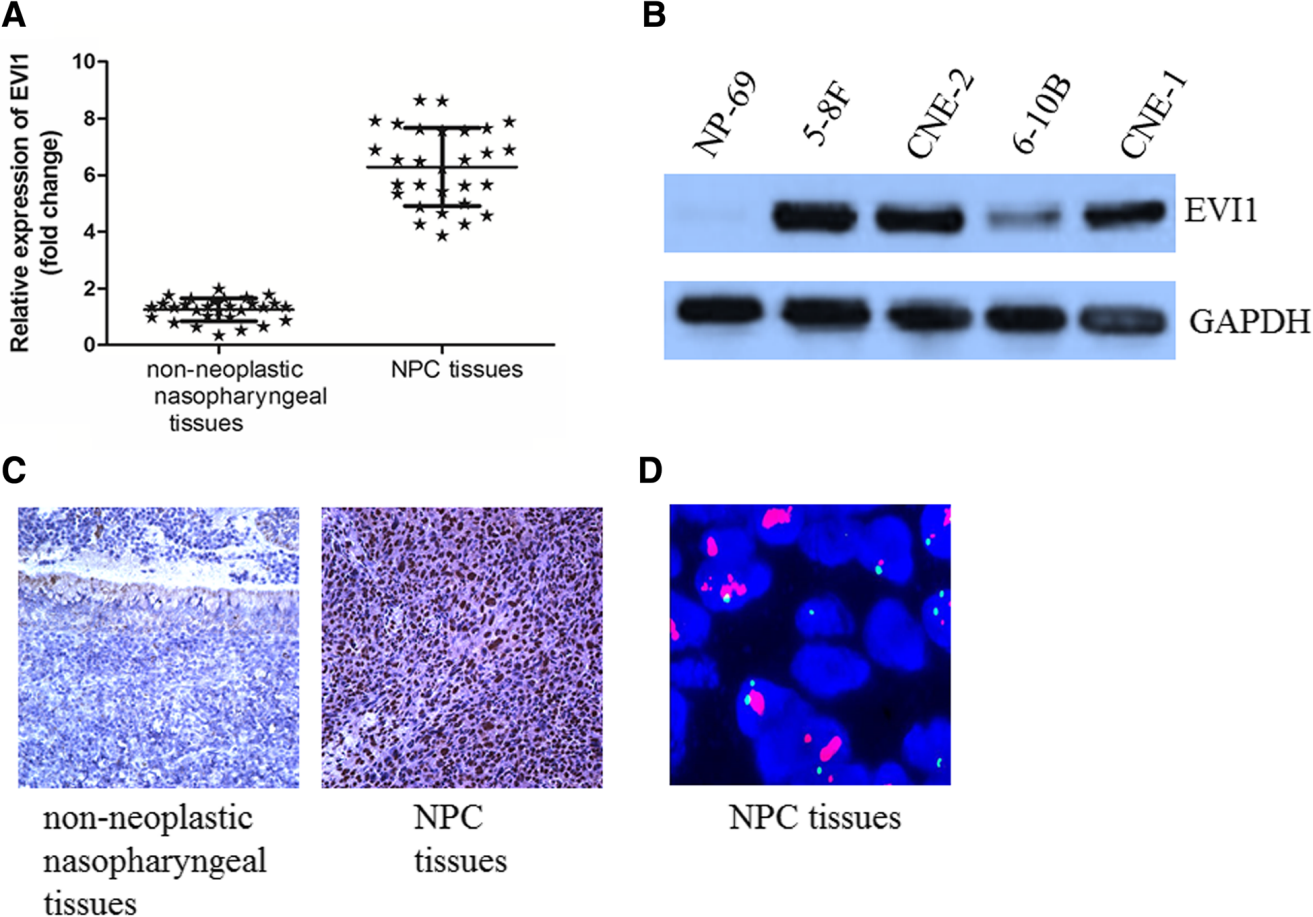
EVI1 expression was elevated in NPC tissues and cell lines (**a**) EVI1 expression was elevated in NPC tissues compared with that in adjacent nonneoplastic nasopharyngeal tissues. (**b**) The expression levels of EVI1 were increased in NPC cell lines. (**c**) EVI1 expression was frequently elevated in NPC tissues. (**d**) EVI1 was amplified in primary NPC tissues

We first examined the expression level of EVI1 in NPC tissues and adjacent nonneoplastic nasopharyngeal tissues. EVI1 expression was found to be higher in NPC tissues than in adjacent nonneoplastic nasopharyngeal tissues (Fig. 1a). In parallel, the expression levels of EVI1 were increased in NPC cell lines (5-8F, CNE1, CNE2 and 6-10B) compared with that in the NP-69 cell line. Among the NPC cell lines, 5-8F cells had the highest expression of EVI1, while 6-10B had the lowest expression of EVI1 (Fig. 1b). An immunohistochemistry (IHC) staining assay revealed that high expression of EVI1 was frequent in primary NPC tissues (89/118, 75.4%) compared with that in nonneoplastic nasopharyngeal tissues (6/50, 12%) (Fig. 1c). In addition, we examined the amplification of EVI1 by fluorescence in situ hybridization in primary NPC tissues (Fig. 1d).

### EVI1 influenced the cell proliferation and invasive capacity of NPC cells in vitro and in vivo

To further clarify the EVI1-mediated molecular events in NPC cells, we conducted a genome-wide analysis to globally characterize EVI1-regulated transcriptome changes. We compared the transcript expression profiles between sh-ctrl and sh-EVI1 cells by using an RNA sequence assay (Additional file 1: Figure S1A and B). A Gene Ontology (GO) assay indicated that the differentially expressed mRNAs were enriched in cell proliferation, cell metastasis, and CSC properties and in drug-resistant signaling pathways (Additional file 1: Figure S1C). In addition, a Gene Set Enrichment Analysis (GSEA) assay showed that EVI1 might play a role in AKT and ERK signaling pathway (Additional file 1: Figure S1D).

**Fig. 2:**
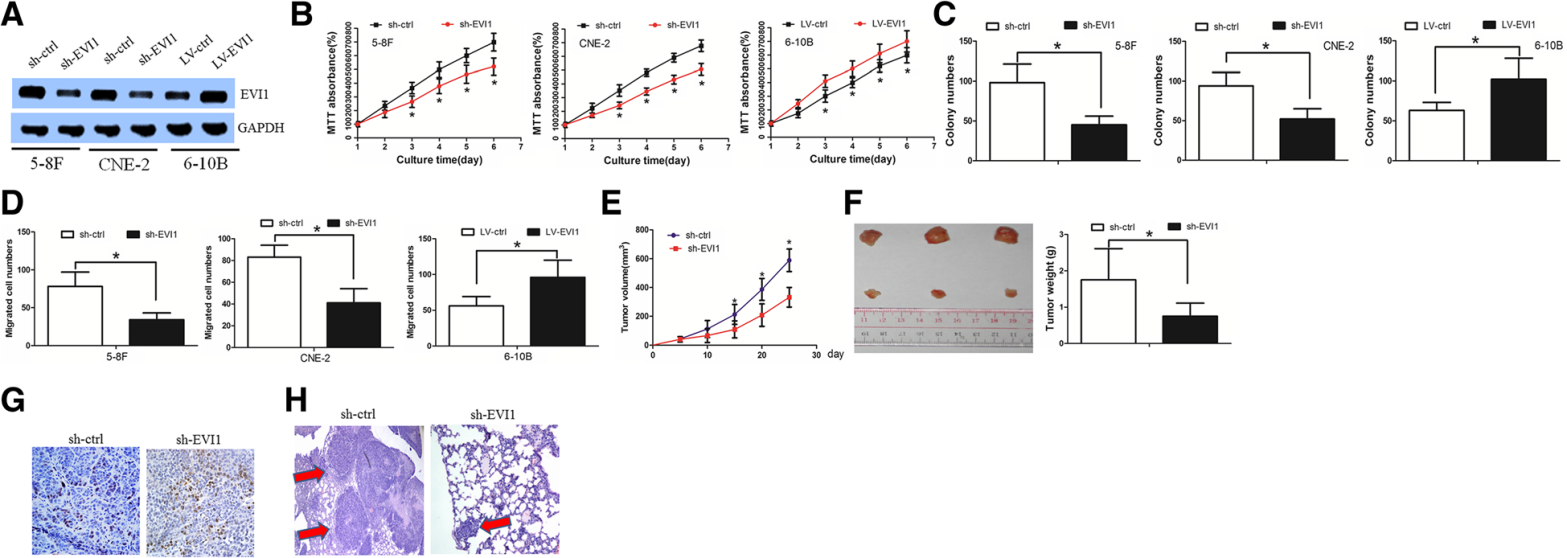
EVI1 influenced the cell proliferation and invasive capacity of NPC cells. (**a**) The EVI1 downregulation and overexpression efficiency was examined by a western blot assay. (**b**) Downregulation of EVI1 significantly decreased the cell proliferation ability, whereas overexpression of EVI1 increased the cell proliferation ability. (**c**) EVI1 downregulation decreased the NPC cell colony formation ability, whereas overexpression of EVI1 increased the cell colony formation ability. (**d**) Downregulation of EVI1 inhibited the invasive ability of 5-8F and CNE-2 cells, while overexpression of EVI1 markedly increased the invasive capacity of 6-10B cells. (**e**) Compared with sh-ctrl cell-derived xenograft tumors, sh-EVI1 cell-derived xenograft tumors grew more slowly. (**f**) The mean weight of sh-EVI1 cell-derived xenograft tumors was also significantly lower than that of sh-ctrl cell-derived xenograft tumors. (**g**) A Ki-67 staining assay revealed that EVI1 downregulation decreased the cell-derived xenograft proliferation rate. (**h**) sh-EVI1 cells formed fewer and smaller nodes per lung than sh-ctrl cells

Based on the above findings, we asked whether EVI1 promoted the cell proliferation and invasive capacity of NPC. We knocked down EVI1 in the 5-8F and CNE-2 cell lines (which had the highest level of endogenous EVI1) and overexpressed EVI1 in the 6-10B cell line (which had the lowest level of endogenous EVI1) (Fig. 2a). Downregulation of EVI1 significantly decreased cell proliferation, whereas overexpression of EVI1 increased cell proliferation as revealed by an MTT assay (Fig. 2b). In addition, EVI1 downregulation decreased the ability of NPC to form cell colonies, whereas overexpression of EVI1 increased cell colony formation (Fig. 2c, Additional file 2: Figure S2A). Downregulation of EVI1 inhibited the invasive ability of 5-8F and CNE-2 cells, while overexpression of EVI1 markedly increased the invasive capacity of 6-10B cells, as determined by Matrigel invasion assays (Fig. 2d).

Subsequently, we performed an in vivo study to analyze the role of EVI1 in cell growth and invasion. A tumorigenesis study was performed to confirm the growth inhibitory effect of EVI1 downregulation in vivo. 5-8F cells treated with sh-ctrl or sh-EVI1 cells were inoculated into the back of nude mice. Compared with sh-ctrl cell-derived xenograft tumors, sh-EVI1 cell-derived xenograft tumors grew more slowly (Fig. 2e). The mean weight of sh-EVI1 cell-derived xenograft tumors was also significantly lower than that of sh-ctrl cell-derived xenograft tumors (Fig. 2f). Next, the tumor sections were stained for Ki-67 expression to quantitatively assess the proliferation index in xenograft tumors (Fig. 2g). These results suggested that EVI1 downregulation exerted an inhibitory effect on the tumorigenesis of NPC cells in vivo. An experimental metastatic assay was then performed to investigate the in vivo effect of EVI1 knockdown on NPC metastasis. 5-8F cells treated with sh-ctrl or sh-EVI1 were injected into the lateral tail vein of severe combined immunodeficient (SCID) mice. The sh-EVI1 cells formed fewer and smaller nodes per lung than the sh-ctrl cells (Fig. 2h).

Taken together, these data suggest that EVI1 is involved in the control of NPC proliferation and metastasis both in vitro and in vivo.

### EVI1 regulated E-cadherin expression via snail in NPC cells

**Fig. 3:**
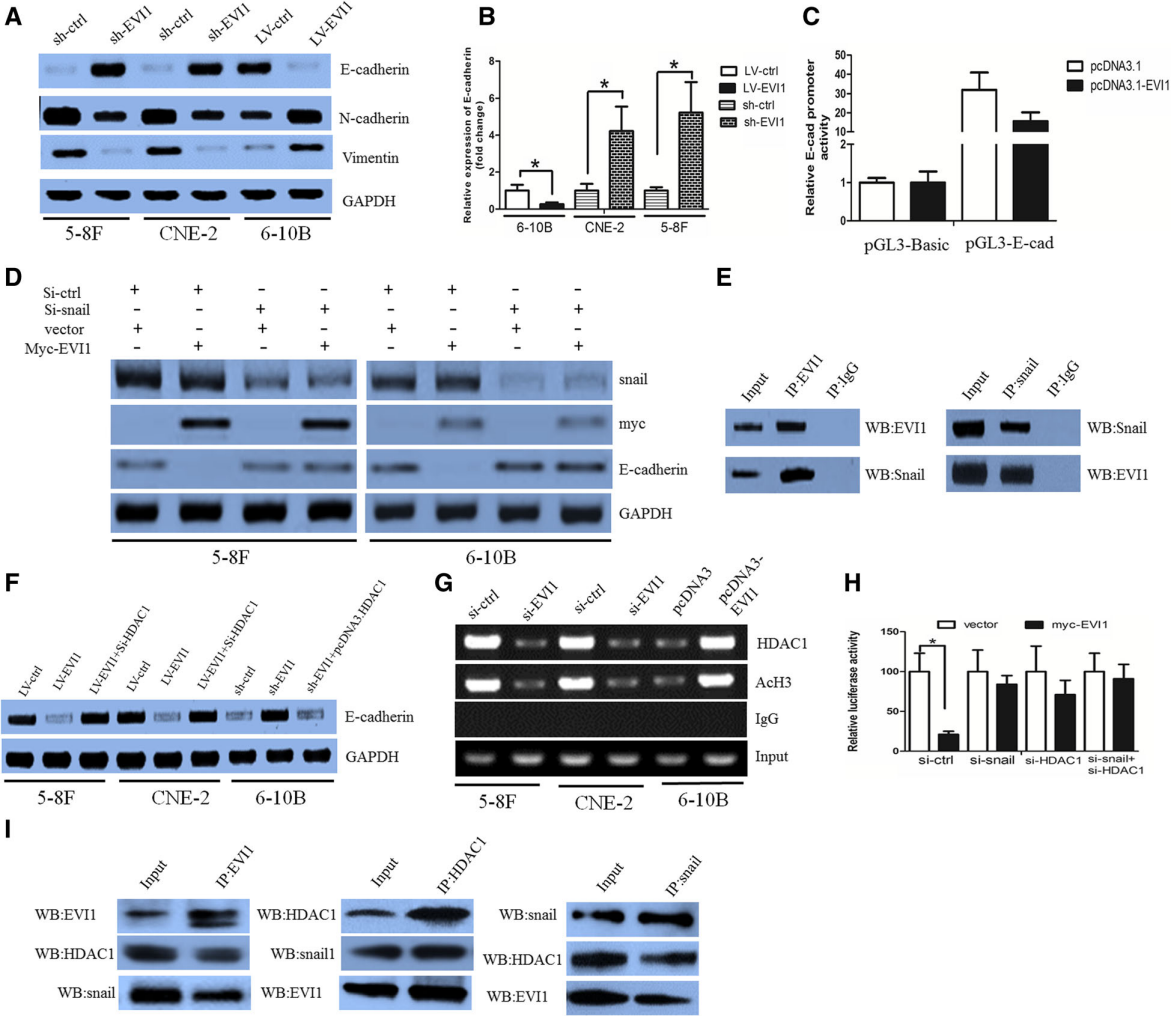
The EVI1/Snail/HDAC1 complex corepressed E-cadherin expression. (**a**) Knockdown of EVI1 elevated E-cadherin expression but decreased N-cadherin and vimentin in 5-8F and CNE-2 cells. Overexpression of EVI1 had the opposite effects in 6-10B cells. (**b**) EVI1 negatively regulated E-cadherin mRNA expression. (**c**) EVI1 suppressed E-cadherin promoter activity in 6-10B cells. (**d**) The suppressive effect of EVI1 on E-cadherin expression was abolished when snail was absent. (**e**) EVI1 physically interacted with snail protein. (**f**) EVI1 downregulation-induced E-cadherin elevation was abolished when HDAC1 was overexpressed (left panel). EVI1-induced repression of E-cadherin expression was restored when HDAC1 was downregulated (right panel). (**g**) When EVI1 was overexpressed in 6-10B cells, the binding of HDAC1 to the E-cadherin promoter was enhanced, and acetylation of histones was decreased. (**h**) Suppressive activity of EVI1 in the E-cadherin promoter of sh-EVI1–5-8F cells was significantly inhibited after the knockdown of endogenous snail or/and HDAC1. (**i**) The existence of snail and HDAC1 was detected in immunoprecipitates obtained with antibody against EVI1 (left panel). EVI1 and snail were detected in HDAC1 immunoprecipitates (middle panel). EVI1 and HDAC1 were detected in snail immunoprecipitates (right panel)

Since the EMT phenotype contributes to cancer metastasis, we asked whether EVI1 regulates the EMT phenotype. 5-8F and CNE-2 cells were two NPC cell lines which have undergone EMT phenotype. The morphology change of the two cell lines was indicated in Additional file 2: Figure S2A. Indeed, knockdown of EVI1 elevated E-cadherin expression but decreased N-cadherin and vimentin. Overexpression of EVI1 had the opposite effects (Fig. 3a).

Loss of E-cadherin is considered a fundamental event in EMT. Loss of E-cadherin promotes cancer metastasis via multiple downstream transcriptional pathways [8]. We thus asked how EVI1 regulates E-cadherin. EVI1 regulated E-cadherin expression not only at the protein level (Fig. 3a) but also at the mRNA level (Fig. 3b, Additional file 2: Figure S2B). These data suggested that EVI1 regulation of E-cadherin occurred at the transcriptional level. We further designed a luciferase reporter construct (contains the 5′-flanking region of the E-cadherin gene) to determine whether EVI1 could downregulate E-cadherin promoter activity. EVI1 suppressed E-cadherin promoter activity when 6-10B cells were cotransfected with pcDNA3.1-EVI1 and pGL3-E-cadherin promoters (Fig. 3c).

To explore the precise molecular mechanisms and relevant interactions with EVI1, mass spectrometry was used in 5-8F cells, and this analysis yielded a list of potential EVI1-interacting proteins (Additional file 2: Figure S2C). Snail was one of the predicted proteins that interacted with EVI1.

A previous study demonstrated that snail is a master transcriptional repressor of E-cadherin [28, 29]. We hypothesized that EVI1 may recruit snail to silence E-cadherin. To confirm our hypothesis, the basic levels of endogenous snail were knocked down by specific small interfering RNA (siRNA) in EVI1-low-expressed 6-10B cells and sh-EVI1–5-8F cells. Then, myc-EVI1 was transfected into these cells to examine whether EVI1 could still repress E-cadherin expression in the absence of snail. Interestingly, the suppressive activity of EVI1 on E-cadherin expression was abolished when snail was absent as determined by the western blot assay (Fig. 3d).

To identify the direct interaction between EVI1 and snail, a coimmunoprecipitation assay was performed in LV-EVI1–6-10B cells. It was revealed that EVI1 physically interacts with snail protein (Fig. 3e). The confocal immunofluorescence assay was performed to confirm the subcellular localization of EVI1 and snail in LV-EVI1–6-10B cells. The nuclear colocalization of EVI1 and snail proteins was clearly detected (Additional file 2: Figure S2D).

Taken together, these data indicated that snail was indispensable for the suppressive effect of EVI1 on E-cadherin.

### The EVI1/snail/HDAC1 complex corepressed the E-cadherin expression

EVI1 has been suggested to associate with HDAC1, a histone deacetylase that plays an important role in the transcriptional repression of E-cadherin [30]. In addition, our data revealed that HDAC1 interacted with EVI1 in NPC cells (Additional file 2: Figure S2C). We reasoned that the EVI1–HDAC1 interaction may also contribute to the repression of E-cadherin.

In sh-EVI1–5-8F cells, EVI1 downregulation-induced E-cadherin elevation was abolished when HDAC1 was overexpressed (Fig. 3f, left panel). In LV-EVI1–6-10B cells, the EVI1-induced repression of E-cadherin expression was restored when HDAC1 was downregulated by siRNA (Fig. 3f, right panel). When EVI1 was knocked down in 5-8F cells, the binding of HDAC1 to the E-cadherin promoter was abolished, and the acetylation of histones was increased (Fig. 3g, left panel). When EVI1 was overexpressed in 6-10B cells, the binding of HDAC1 to the E-cadherin promoter was enhanced, and the acetylation of histones was decreased (Fig. 3g, right panel). Taken together, these data indicated that EVI1 may repress E-cadherin expression via the recruitment of HDAC1 to the E-cadherin promoter.

Subsequently, a luciferase reporter assay was used to examine whether the suppressive effect of EVI1 on the E-cadherin promoter requires both snail and HDAC1. Sh-EVI1–5-8F cells were transfected with specific siRNAs targeted to snail and HDAC1. Subsequently, these cells were reintroduced with the myc-EVI1 plasmid, and the luciferase activity of the E-cadherin promoter was measured. The suppressive activity of EVI1 in the E-cadherin promoter of sh-EVI1–5-8F cells was significantly inhibited after the knockdown of endogenous snail but was only partially inhibited after the downregulation of HDAC1 (Fig. 3h). Compared with treatment with each siRNA alone, a combined treatment with si-HDAC1 and si-snail caused the repressive activity to be substantially enhanced (Fig. 3h).

We performed a coimmunoprecipitation assay to examine whether EVI1, snail and HDAC1 could interact with each other in 5-8F cells. As shown in Fig. 3i (left panel), the presence of snail and HDAC1 was detected in immunoprecipitates obtained with antibody against EVI1. In parallel, EVI1 and snail were detected in HDAC1 immunoprecipitates (Fig. 3i, middle panel). EVI1 and HDAC1 were detected in snail immunoprecipitates (Fig. 3i, right panel).

Consequently, we hypothesized that EVI1, snail, and HDAC1 formed a corepressor complex to repress E-cadherin expression.

### EVI1 regulated CSC properties in NPC

**Fig. 4:**
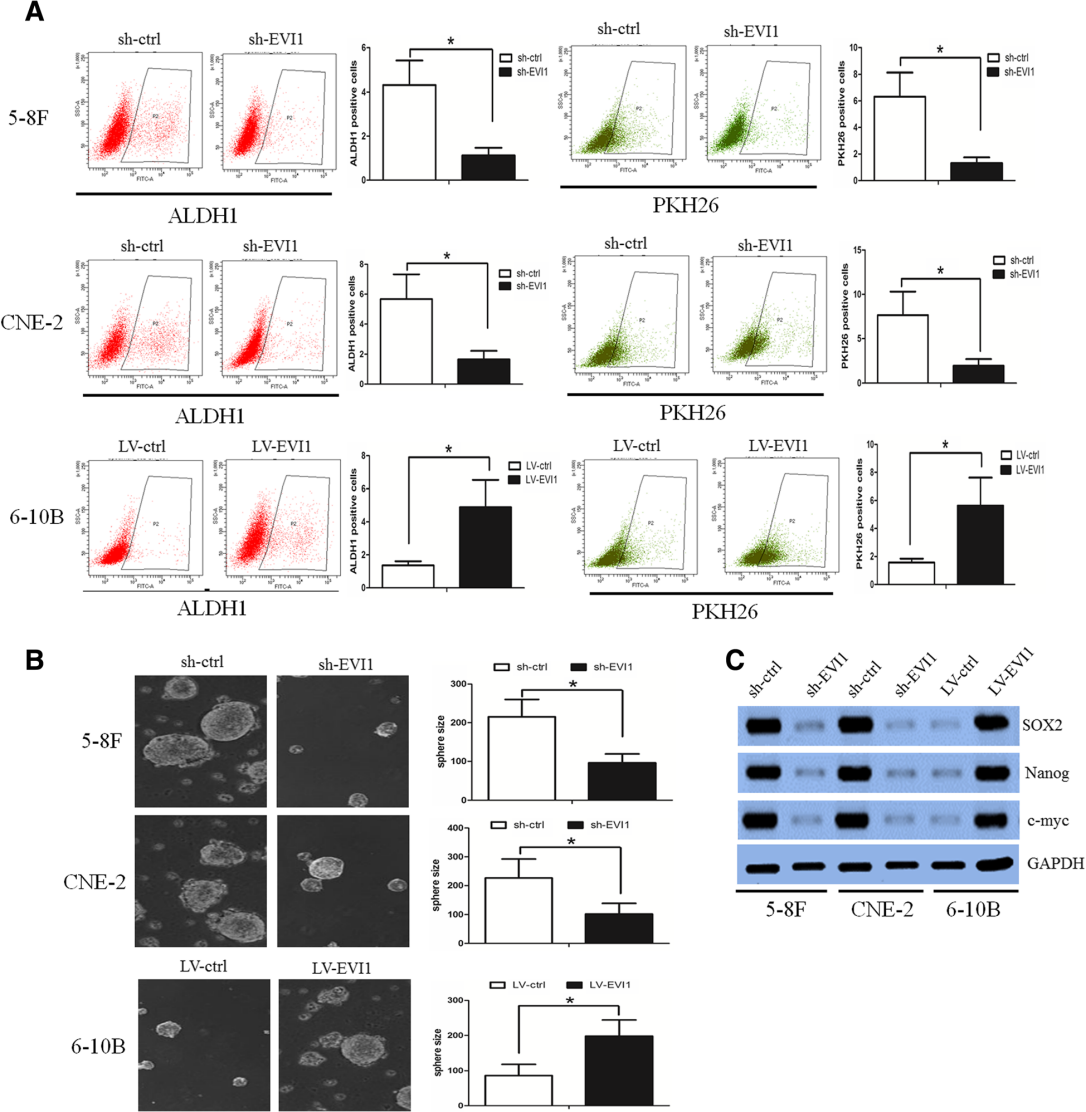
EVI1 regulated CSC properties in NPC cells. (**a**) PKH26 + − and ALDH1 + −labeled cells were significantly decreased in sh-EVI1–5-8F and sh-EVI1-CNE-2 cells compared with those in sh-ctrl cells. However, when EVI1 was overexpressed in 6-10B cells, the PKH26 + − and ALDH1 + −labeled cells were significantly increased. (**b**) The sphere formation ability was impaired in sh-EVI1–5-8F and sh-EVI1-CNE-2 cells but was enhanced in LV-EVI1–6-10B cells. (**c**) The expression levels of SOX2, Nanog and c-myc were decreased in sh-EVI1–5-8F and sh-EVI1-CNE-2 cells but were enhanced in LV-EVI1–6-10B cells

Our previous study revealed that PKH26+ and ALDH1+ NPC cells had CSC properties [14, 15]. We found that PKH26 + −labeled and ALDH1 + −labeled cells were significantly decreased among sh-EVI1–5-8F and sh-EVI1-CNE-2 cells compared with those among sh-ctrl cells (Fig. 4a). However, when EVI1 was overexpressed in 6-10B cells, PKH26 + − and ALDH1 + −labeled cells were significantly increased (Fig. 4a). In parallel, the sphere formation ability was impaired in sh-EVI1–5-8F and sh-EVI1-CNE-2 cells (Fig. 4b) but was enhanced in LV-EVI1–6-10B cells (Fig. 4b). The pluripotent transcription factors (SOX2, Nanog, c-Myc, and Oct4) were implicated in the self-renewal of CSCs, including in NPC CSCs [31]. Our data revealed that the expression levels of SOX2, Nanog and c-myc were decreased in sh-EVI1–5-8F and sh-EVI1-CNE-2 cells (Fig. 4c) but were enhanced in LV-EVI1–6-10B cells (Fig. 4c). These data suggested that EVI1 regulated CSC properties in NPC.

### EVI1 regulated the Wnt/β-catenin signaling pathway in NPC

**Fig. 5:**
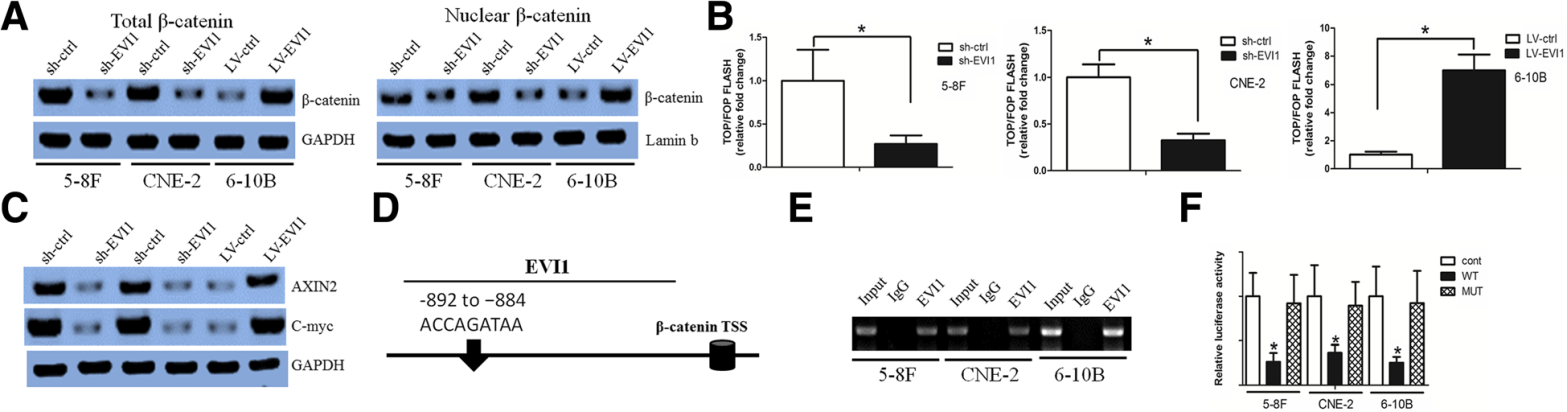
EVI1 regulated the Wnt/β-catenin signaling pathway in NPC. (**a**) EVI1 downregulation inhibited β-catenin expression in the total cell lysate and the nuclear fraction. In contrast, forced expression of EVI1 enhanced β-catenin expression in the total cell lysate and the nuclear fraction. (**b**) The Wnt/β-catenin activity was repressed when EVI1 was knocked down as revealed by a TOP/FOP Flash reporter assay (left panel). EVI1 elevated Wnt/β-catenin activity when EVI1 was overexpressed (right panel). (**c**) The expression levels of β-catenin downstream targets AXIN2 and c-myc were downregulated in sh-EVI1 cells and were upregulated in LV-EVI1 cells. (**d**) The β-catenin promoter region contains one putative EVI1-binding site. (**e**) A ChIP assay demonstrated that EVI1 bound to the β-catenin promoter. (**f**) EVI1 positively regulated the β-catenin promoter activity

Our previous study demonstrated that the Wnt/β-catenin signaling pathway regulated CSC properties, including those in NPC [32–34]. We thus asked whether EVI1 exerted its function in CSCs via the Wnt/β-catenin signaling pathway. EVI1 downregulation inhibited β-catenin expression in the total cell lysate and in the nuclear fraction. In contrast, forced expression of EVI1 enhanced β-catenin expression in the total cell lysate and in the nuclear fraction (Fig. 5a). Consistent with these results, the Wnt/β-catenin activity was repressed when EVI1 was knocked down as revealed by a TOP/FOP Flash reporter assay (Fig. 5b). In addition, EVI1 overexpression elevated the Wnt/β-catenin activity (Fig. 5b), and the expression levels of the β-catenin downstream targets AXIN2 and c-myc were downregulated in sh-EVI1 cells and upregulated in LV-EVI cells (Fig. 5c). We then performed rescue experiments through the stable expression of constitutively active β-catenin in sh-EVI1 cells and knocked down β-catenin in LV-EVI1 cells to confirm that the stemness properties induced by EVI1 were mediated through the Wnt/β-catenin signaling pathway. The enforced expression and si-β-catenin were confirmed by a western blot assay (Additional file 2: Figure S2E), and the functional effects on CSCs upon silencing or overexpression of EVI1 were partially rescued by the forced expression of β-catenin or si-β-catenin, respectively ((Additional file 2: Figure S2F-J).

### EVI1 regulated β-catenin expression by binding to its promoter

We used LASAGNA-Search 2.0 to determine that the β-catenin promoter region contained one putative EVI1-binding site (Fig. 5d). A chromatin immunoprecipitation (ChIP) assay was used to determine whether EVI1 binds to the β-catenin promoter in 5-8F and 6-10B cells. In addition, we found significant enrichment of this specific region from immunoprecipitated chromatin DNA when compared to negative control (IgG) pulldown (Fig. 5e). Subsequently, wild-type and mutant β-catenin promoters were cloned into the pGL3-Basic vector to examine the effect of EVI1 on promoter activity. The wild-type and mutant promoters were cotransfected with EVI1 plasmid into 5-8F and 6-10B cells. We observed that the luciferase activities of the wild-type promoter, instead of the mutant promoter, significantly increased when EVI1 was cotransfected (Fig. 5f). These data indicated that EVI1 increased the transcriptional activity of the β-catenin promoter.

To further clarify whether EVI1 exerts oncogenic activities via WNT signaling pathway, we tested the efficacy of WNT inhibitor drug Cardamonin (CAS 19309–14-9) in nasopharyngeal carcinoma cell lines (5-8F, CNE-2 and LV-EVI1–6-10B). We revealed that CAS 19309–14-9 inhibited growth and invasion in these cell lines (Additional file 3: Figure S4A-C). Interestingly, Wnt agonist drug CAS 853220–52-7 reinforced tumorigenicity in EVI1 knockdown cells (sh-EVI1–5-8F and sh-EVI1-CNE2) (Additional file 3: Figure S4D).

### EVI1 upregulation predicted an unfavorable prognosis and contributed to chemo−/radioresistance in NPC cells

**Fig. 6:**
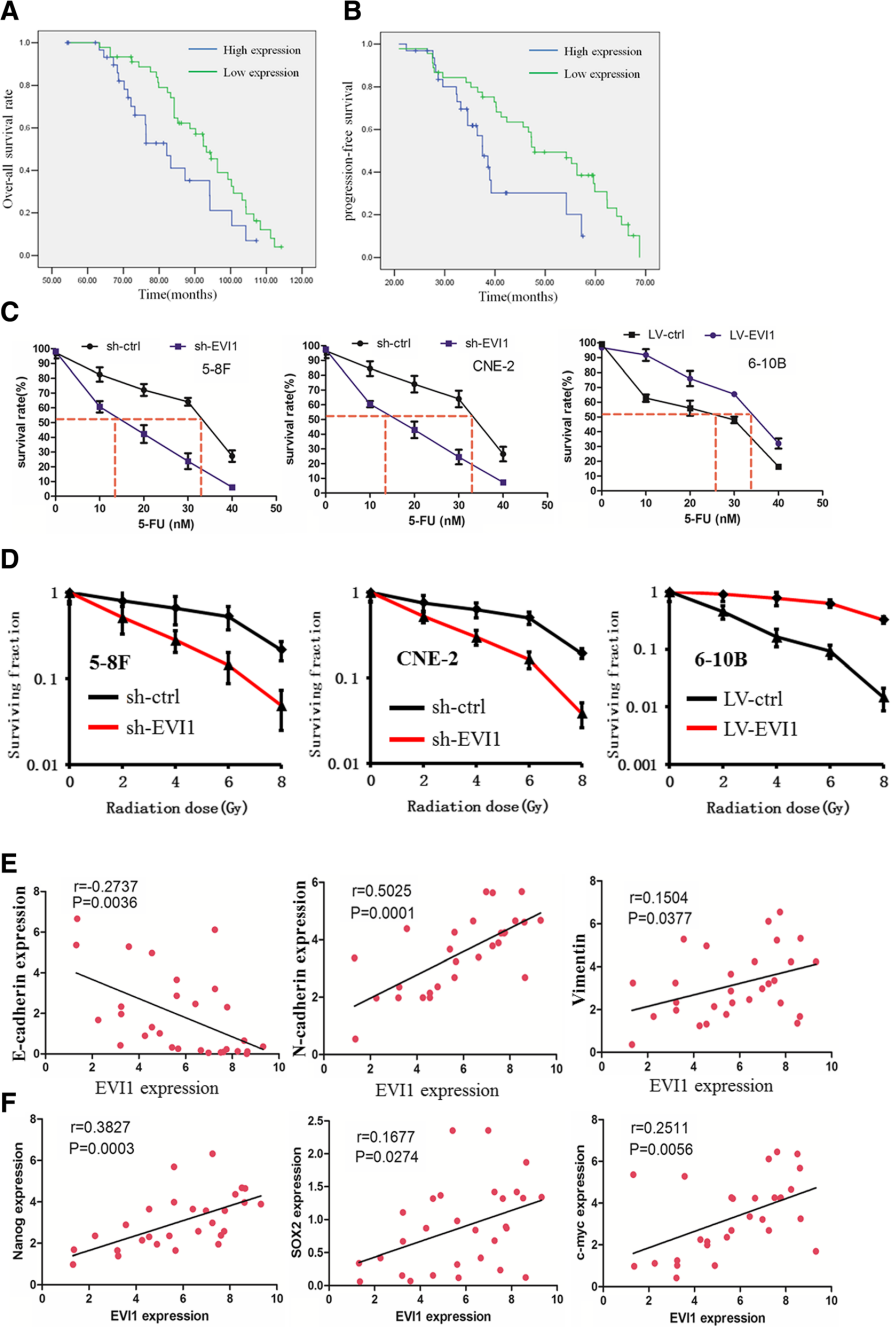
EVI1 upregulation predicted an unfavorable prognosis and contributed to chemo−/radioresistance in NPC cells. (**a**) High-level EVI1 expression was correlated with a shorter OS rate. (**b**) High-level EVI1 expression was correlated with a shorter PFS rate. (**c**) Knockdown of EVI1 increased NPC cell sensitivity to 5-Fu, while overexpression of EVI1 decreased NPC cell sensitivity to 5-Fu as revealed by an MTT assay. (**d**) EVI1 downregulation increased NPC cell radiosensitivity, while overexpression of EVI1 decreased the NPC cell sensitivity to irradiation as revealed by an MTT assay. (**e**) EVI1 expression was negatively associated with E-cadherin expression, but positively associated with N-cadherin and Vimentin expression.(**f**) expression was positively associated with Nanog, SOX2 and c-myc expression

Next, we explored the association between EVI1 expression and NPC patient prognosis. We present the associations between EVI1 expression and patient clinicopathological features in Table 2. Parameters such as gender (*P* = 0.121), age (*P* = 0.122) and EBV infection (*P* = 0.108) were not significantly associated with EVI1 expression. However, high EVI1 expression was significantly correlated with tumor size (*P* = 0.011), lymph node metastasis (*P* = 0.002), distant metastasis (*P* = 0.000) and late clinical stage (P = 0.011). In the Kaplan-Meier analysis assay, we found that high-level EVI1 expression was correlated with shorter overall survival (OS) and progression-free survival (PFS) (Fig. 6a and b, *P* < 0.05).

**Table 2:** Correlation of EVI1 expression in tissue with patients clinicopathological variables in NPC

	EVI1			
Variables	All case (*n* = 98)	Low expression (*n* = 35)	High expression (*n* = 63)	*P* Value*
Age(years)				
< 60	38	10	28	0.122
≧60	60	25	35	
Sex				
Male	69	28	41	0.121
Female	29	7	22	
Smoking				
Yes	59	24	35	0.207
No	39	11	28	
EBV				
Positive	66	20	46	0.108
Negative	32	15	17	
T classification				
T1-T2	42	9	33	0.011*
T3-T4	56	26	30	
N classification				
N0-N1	58	28	30	0.002*
N2-N3	40	7	33	
M classification				
M0	46	27	19	0.000*
M1	52	8	44	
TNM clinical stage				
I, II	59	27	32	0.011*
III, IV	39	8	31	

The symbol * means statistically significant

We then examined the effect of EVI1 on chemo−/radioresistance. Sh-ctrl, sh-EVI1, LV-ctrl and LV-EVI1 cells were treated with different doses of 5-Fu, and cell viability was examined by an MTT assay. We found that knockdown of EVI1 increased NPC cell sensitivity, while overexpression of EVI1 decreased NPC cell sensitivity to 5-Fu (Fig. 6c). Subsequently, sh-ctrl, sh-EVI1, LV-ctrl and LV-EVI1 cells were treated with different doses of radiation. Similarly, EVI1 downregulation increased the NPC cell radiosensitivity, while overexpression of EVI1 decreased the NPC cell sensitivity to irradiation as revealed by the MTT assay (Fig. 6d). These data indicated that EVI1 conferred chemo−/radioresistance in NPC cells.

### Correlation of EVI1 with EMT and CSC-related genes

We then analyzed the correlation of EVI1 with EMT and CSC-related genes. In EMT-related genes, EVI1 expression was negatively associated with E-cadherin expression (Fig. 6e, Additional file 4: Figure S3K) but positively associated with N-cadherin and vimentin expression (Fig. 6e, Additional file 2: Figure S2K). In CSC-related genes, EVI1 expression was positively associated with SOX2, c-myc and Nanog expression (Fig. 6f, Additional file 2: Figure S2K).

### Arsenic trioxide exerted an antitumor effect in NPC cells

Arsenic trioxide (ATO) has been reported to degrade the oncogenic EVI1 protein [35], and thus, we further asked whether ATO exerts an antitumor effect in NPC cells. We chose the 5-8F and CNE-2 cell lines (which had the highest expression levels of EVI1) for further study. We first used folate (FA)-labeled human serum albumin (HSA) to load ATO and form arsenite-loaded nanoparticles (ALNPs). Transmission electron microscopy (TEM) imaging revealed that the ALNPs were uniform in size distribution, with core-shell nanostructures (Additional file 4: Figure S3A). The size of the ALNPs was approximately 50–60 nm as determined by dynamic light scattering (DLS) (Additional file 4: Figure S3B). To validate the efficiency at the cellular level, we determined the cellular uptake of FITC-labeled ALNPs by fluorescence microscopy. We performed an MTT assay to evaluate the half-maximal inhibitory concentration of ALNPs and free ATO in NPC cells and found that the ALNP drug delivery system, compared with free ATO, significantly elevated the cytotoxicity to NPC cells (Additional file 4: Figure S3C). In addition, we found that ALNPs degraded the EVI1 protein in these cell lines (Additional file 4: Figure S3D).

**Fig. 7:**
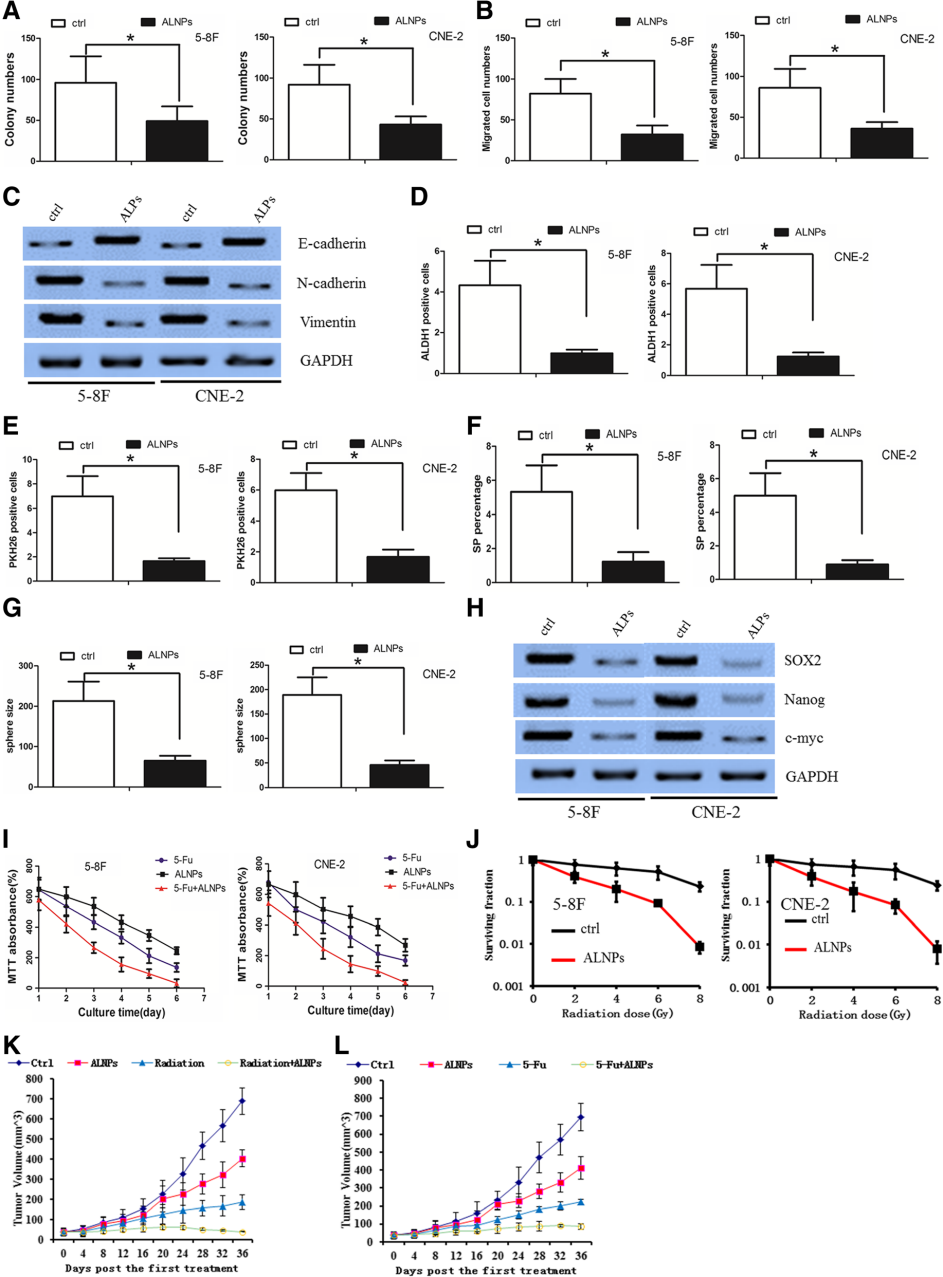
ALNPs exerted an antitumor effect in NPC cells. (**a**) ALNPs decreased the NPC cell colony formation ability. (**b**) ALNPs decreased the NPC cell migration ability. (**c**) ALNPs increased E-cadherin expression but decreased N-cadherin and vimentin expression. (**d**) ALNPs decreased the ALDH1+ labeling cell rate. (**e**) ALNPs decreased the PKH26+ labeling cell rate. (**f**) ALNPs decreased the SP rate. (**g**) ALNPs decreased the tumor sphere size. (**h**) ALNPs decreased the CSC-related markers SOX2, Nanog and c-myc. (**i**) ALNPs increased the NPC cell sensitivity to 5-Fu, as determined by an MTT assay. (**j**) ALNPs increased the NPC cell sensitivity to radiation, as determined by an MTT assay. (**k**) ALNPs increased the NPC cell sensitivity to 5-Fu in vivo. (**l**) ALNPs increased the NPC cell sensitivity to radiation in vivo

ALNP treatment inhibited NPC colony formation and invasion ability in vitro (Fig. 7a-b). A western blot assay revealed that ALNPs increased E-cadherin expression and decreased N-cadherin and vimentin expression (Fig. 7c). In addition, ALNPs eliminated CSC activity in NPC cells (Fig. 7d-g). The CSC-related markers were decreased in ALNP-treated NPC cells (Fig. 7h). Interestingly, ALNPs also increased NPC cell sensitivity to 5-Fu and radiation (Fig. 7i-j). These data suggest that ALNPs mimicked the effect of EVI1 downregulation on NPC cells in vitro. We also tested whether ATO still had an effect on EVI1 knockdown cells. We found that ATO treatment had limited effect on EVI1 knockdown cells(sh-EVI1–5-8F and sh-EVI1-CNE-2 cells)(data not shown). However, in cell line LV-EVI1–6-10B, EVI-1 overexpression effect on cell growth and invasion could be partly counteracted by ATO treatment(Additional file 3: Figure S4E). These data suggest that ATO exert its function on NPC mainly on EVI1.

Finally, we analyzed the antitumor effect of ALNPs and found that these nanoparticles decreased the tumor growth in vivo. Interestingly, when combined with 5-Fu or radiation treatment, ALNPs had synergistic effects with both 5-Fu and radiation (Fig. 7k-l, Additional file 4: Figure S3E-F). To evaluate the systemic animal toxicity of ALNPs, the main organ tissues of mice were subjected to H&E staining. We observed that all the organs maintained their typical structures and did not exhibit appreciable microscopic lesions (Additional file 4: Figure S3G). These data suggested that ALNPs may be an effective biosafety treatment.

## Discussion

Previous studies have well documented the abnormal expression and biological role of EVI1 in a series of cancers [36, 37]. As an oncogene located on chromosome 3q26 [38], EVI1 is amplified in NPC tissues [39]. A recent study suggested that EVI1 may be involved in NPC tumorigenesis [40]. However, the biological role and the underlying mechanism of EVI1 in NPC have seldom been reported.

The current study revealed that the EVI1 expression level was upregulated in NPC tissues and cell lines. The reasons for the high expression of EVI1 differ in various cancers. Genetic rearrangements of the EVI1 locus at chromosomal region 3q26 contribute to the elevated expression of EVI1 in myeloid leukemia [41]; however, high copy number gains were the main cause of abnormal EVI1 expression levels in ovarian cancer [42]. In addition, alternative mechanisms may also lead to the aberrant expression of EVI1 [41]. We found that DNA amplification contributed to the elevated expression of EVI1 in NPC as revealed by a fluorescence in situ hybridization assay. This result was consistent with previous findings [40].

Other reports have documented that EVI1 promotes cell proliferation, migration and metastasis [23, 43–45]. Consistent with these results, our data demonstrated that EVI1 contributed to NPC cell growth and invasion both in vitro and in vivo. These findings indicated that EVI1 may function as an oncogene in NPC. Loss of E-cadherin is regarded to enable metastasis by disrupting intercellular contacts—an early step in metastatic dissemination [8]. Previous research found that EVI1 negatively regulates E-cadherin [36, 46]. However, the underlying mechanism is still poorly understood. In our study, we used a mass spectrometry assay to identify snail and HDAC1 as two important proteins associated with EVI1. Previous documents have demonstrated that snail and epigenetic modulator HDACs play significant roles in the metastasis of carcinomas. Oncogenes can interact with snail and HDACs, form a multiprotein complex and thereby inhibit E-cadherin expression. For instance, Nie et al. showed that the hepatitis C virus core protein recruits snail and HDAC1/HDAC2 to the E-cadherin promoter and decreases E-cadherin expression [47]. Tong et al. reported that EZH2 inhibits E-cadherin expression by forming a corepressor complex with HDAC1/HDAC2 and snail [48]. Concordantly, we found that EVI1 recruited snail and HDAC1 to decrease E-cadherin expression. Notably, EVI1 may directly target PTEN and regulate PI3K/AKT activity [49]. Our GSEA model revealed that EVI1 may also regulate the PI3K/AKT pathway. Thus, EVI1 might repress E-cadherin expression via the PTEN/ PI3K/AKT pathway. Subsequently, we showed that EVI1 elevated N-cadherin and vimentin expression in NPC cells. Overexpression of EVI1 caused a spindle shape and increased lamellipodia in NPC cells. We speculated that EVI1 induced an EMT phenotype and thus promoted NPC cell metastasis. Our findings conflicted with those of a previous study [36], which demonstrated that EVI1 is dispensable for EMT but is required for metastasis. This may be the reason that EVI1 exerted its function in different cell contexts.

Cancer cells that undergo EMT can generate CSCs [50]. A previous study revealed that EVI1 contributes to prostate cancer CSC formation and thus promotes prostate cancer initiation [45]. In chronic myeloid leukemia, Evi1-high leukemic cells possessed a greater potential than EVI1-low cells in oncogenic self-renewal, and EVI1 was a functional marker of stem cells [51]. However, how EVI1 regulates CSC properties is still poorly understood.

Our previous study revealed that PKH26+ and ALDH1+ NPC cells display CSC properties [14, 15]. We demonstrated that EVI1 downregulation decreased the PKH26+ and ALDH1+ cell rate, while overexpression of EVI1 elevated the PKH26+ and ALDH1+ cell rate. In parallel, EVI1 elevated the SP rate and tumor sphere size. The CSC biomarkers c-myc, Nanog and SOX2 were elevated when EVI1 was overexpressed but were decreased when EVI1 was knocked down. We further explored the underlying mechanism of how EVI1 regulated CSC properties. We identified that EVI1 directly bound to the β-catenin promoter. β-catenin signaling was important for maintenance of the CSC phenotype. Our previous studies revealed that β-catenin regulated the CSC properties in a series of cancers, including NPC [32–34]. When binding with cadherin at the membrane, β-catenin controls cell-cell adhesion. However, when activated by stimulators, β-catenin acts as a transcriptional activator in the nucleus, where it interacts with LEF1 and TCF transcription factors [52]. Our previous study revealed that nuclear β-catenin is responsible for the maintenance of CSCs properties [32–34].

Our present data revealed that EVI1 increased the transcriptional activity of the β-catenin promoter and promoted β-catenin nuclear translocation in NPC cells. β-catenin downregulation blocked the effects of EVI1 on CSC properties. In summary, our data confirmed that EVI1 was a functional marker of CSCs and revealed that β-catenin mediated the function of EVI1 in CSCs.

The EMT phenotype, together with CSCs, contributes to chemo−/radioresistance in cancer [53]. Targeting EMT and CSC maintenance is a promising therapeutic strategy in cancer. Our above findings revealed that downregulation of EVI1 reversed the EMT phenotype and eliminated CSC properties. We further asked whether targeting EVI1 could overcome chemo−/radioresistance in NPC cells. As expected, knockdown of EVI1 increased NPC cell sensitivity to 5-Fu and irradiation. A previous study revealed that Evi1 defines tyrosine kinase inhibitor resistance in chronic myeloid leukemia [51]. In acute myeloid leukemia, EVI1 decreased the cell sensitivity to daunorubicin [54]. In solid cancer, knockdown of EVI1 restored prostate cancer sensitivity to docetaxel [45]. These findings are parallel with our data.

In our clinical NPC sample analysis, high-level EVI1 expression was correlated with shorter OS and PFS. These data indicated that EVI1 may be an unfavorable prognosis factor in NPC. The association of EVI1 and EMT and CSC-related genes was also demonstrated in NPC tissues, which further confirmed the in vitro study.

ATO can degrade EVI1 protein and is used for the clinical treatment of acute promyelocytic leukemia [55]. In addition, ATO may have a promising therapeutic effect on solid tumors [56]. However, the low water solubility and adverse side effects of ATO [57] restrict the its efficacy in the treatment of solid tumors. Drug delivery systems based on nanotechnology can improve drug efficacy and reduce side effects [58, 59]. We thus designed HAS-loaded ATO and tested the antitumor effect of this drug system. EVI1 protein was degraded by the ALNP drug system. ALNPs decreased the NPC cell growth and invasion ability effectively. In addition, ALNPs exhibited few side effects in mice, suggesting that HAS-loaded ATO may be effectively and safely used in cancer treatment.

In summary, we demonstrated that EVI1 is elevated in NPC tissues and cell lines. EVI1 may promote the EMT phenotype and CSC properties in NPC cells. EVI1 confers chemo−/radioresistance in NPC cells (wording model as Fig. 8). Treatment with ATO may be an effective option for targeting EVI1 in NPC.

## Conclusions

In conclusion, our study demonstrated that EVI1 was upregulated in NPC tissues and cell lines. EVI1 repressed E-cadherin expression and ultimately contributed to EMT phenotype in NPC cells. β-catenin mediated EVI1’s function on cancer stem cells properties. EVI1 up-regulation predicted unfavorable prognosis and contributed to chemo/radio-resistance in NPC cells. Our findings indicated that EVI1 may be a potential therapeutic target for NPC treatment.

## Additional files

Additional file 1: **Figure S1**. (A)Volcano plot showing the difference in transcripts between the sh-ctrl and sh-EVI1 group. (B) Circle map showing the distribution patterns of different transcripts in the human genome. (C) GO enrichment analysis of EVI1-regulated mRNAs. (D) A GSEA assay showed that EVI1 might play a role in the AKT and ERK signaling pathway. (TIF 5589 kb)

Additional file 2: **Figure S2**. (A) The morphology change of NPC cells which have undergone EMT phenotype(5-8F and CNE-2 cell lines). (B) EVI1 regulated E-cadherin mRNA expression. (C) EVI1-interacting proteins were indicated. (D) Nuclear colocalization of EVI1 and snail proteins was clearly detected in NPC cells. (E) The enforced expression and si-β-catenin were confirmed by western blot assay. (F)- (J) Functional effects on CSCs upon silencing or overexpression of EVI1 were partially rescued by the forced expression of β-catenin or si-β-catenin. (K) The expression of EVI1, E-cadherin, N-cadherin, vimentin, SOX2, Nanog and c-myc in NPC tissues as revealed by an IHC assay. (TIF 12346 kb)

Additional file 3: **Figure S4**. (A) WNT inhibitor drug Cardamonin (CAS 19309–14-9) decreased cell proliferation in 5-8F, CNE-2 and LV-EVI1–6-10B cells, as revealed by MTT assay. (B) WNT inhibitor drug Cardamonin (CAS 19309–14-9) impaired colony formation ability of 5-8F, CNE-2 and LV-EVI1–6-10B cells. (C) The transwell assay revealed that WNT inhibitor drug Cardamonin (CAS 19309–14-9) decreased cell invasion ability of 5-8F, CNE-2 and LV-EVI1–6-10B cells. (D) Wnt agonist drug CAS 853220–52-7 reinforced cell growth, colony formation and invasion ability in sh-EVI1–5-8F and sh-EVI1-CNE-2 cells. (E) EVI-1 overexpression effect on cell growth, colony formation and invasion ability could be partly counteracted by ATO treatment. (TIF 6304 kb)

Additional file 4: **Figure S3**. (A) TEM images revealed that the ALNPs were uniform in size distribution with core-shell nanostructures. (B) The size of ALNPs was approximately 50–60 nm as determined by DLS. (C) Compared with free ATO, the ALNP drug delivery system significantly elevated the cytotoxicity to NPC cells as revealed by an MTT assay. (D) ALNPs degraded the EVI1 protein in NPC cell lines. (E)-(F) ALNPs have synergistic effects with both 5-Fu and radiation. (G) H&E staining of tissue sections from the main organs of mice in the PBS- and ALNP-treated groups. (TIF 7703 kb)

## Abbreviations

ALNPs: arsenic trioxide-loaded nanoparticles
Co-IP: Coimmunoprecipitation
CSC: cancer stem cells
EBV: Epstein-Barr virus
EMSA: Electrophoretic mobility shift assay
EMT: epithelial-to-mesenchymal transition
NPC: nasopharyngeal carcinoma
PVDF: polyvinylidene fluoride
