# Supplementary figures and images for: EVI1 promotes epithelial-to-mesenchymal transition, cancer stem cell features and chemo−/radioresistance in nasopharyngeal carcinoma
Source: J Exp Clin Cancer Res. 2019 Feb 15;38:82. doi: 10.1186/s13046-019-1077-3 (PMC6377731; doi:10.1186/s13046-019-1077-3)

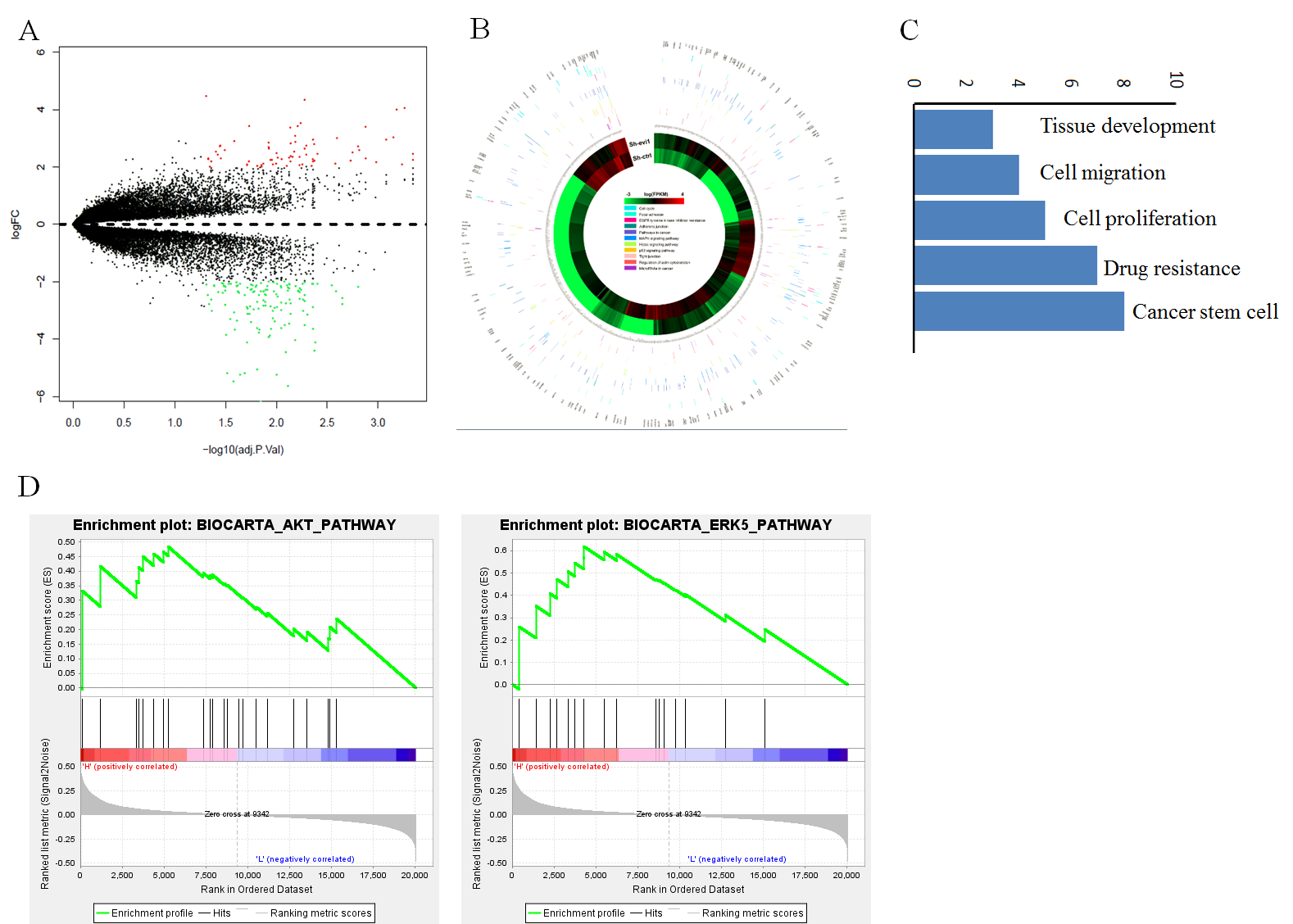

Supplement: Supplementary file 1 — Figure S1. (A)Volcano plot showing the difference in transcripts between the sh-ctrl and sh-EVI1 group. (B) Circle map showing the distribution patterns of different transcripts in the human genome. (C) GO enrichment analysis of EVI1-regulated mRNAs. (D) A GSEA assay showed that EVI1 might play a role in the AKT and ERK signaling pathway. (TIF 5589 kb) [file 13046_2019_1077_MOESM1_ESM.tif]

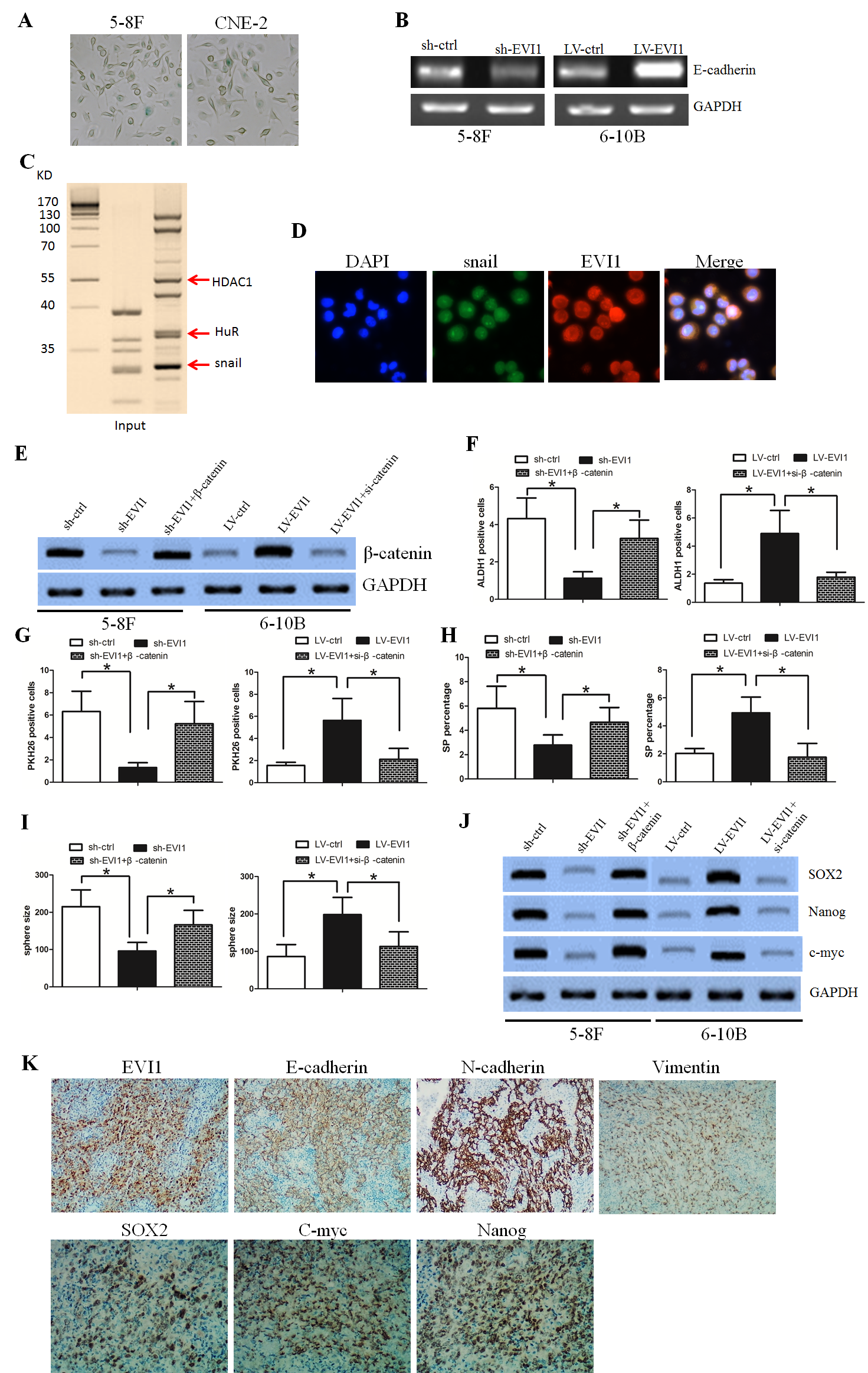

Supplement: Supplementary file 2 — Figure S2. (A) The morphology change of NPC cells which have undergone EMT phenotype(5-8F and CNE-2 cell lines). (B) EVI1 regulated E-cadherin mRNA expression. (C) EVI1-interacting proteins were indicated. (D) Nuclear colocalization of EVI1 and snail proteins was clearly detected in NPC cells. (E) The enforced expression and si-β-catenin were confirmed by western blot assay. (F)- (J) Functional effects on CSCs upon silencing or overexpression of EVI1 were partially rescued by the forced expression of β-catenin or si-β-catenin. (K) The expression of EVI1, E-cadherin, N-cadherin, vimentin, SOX2, Nanog and c-myc in NPC tissues as revealed by an IHC assay. (TIF 12346 kb) [file 13046_2019_1077_MOESM2_ESM.tif]

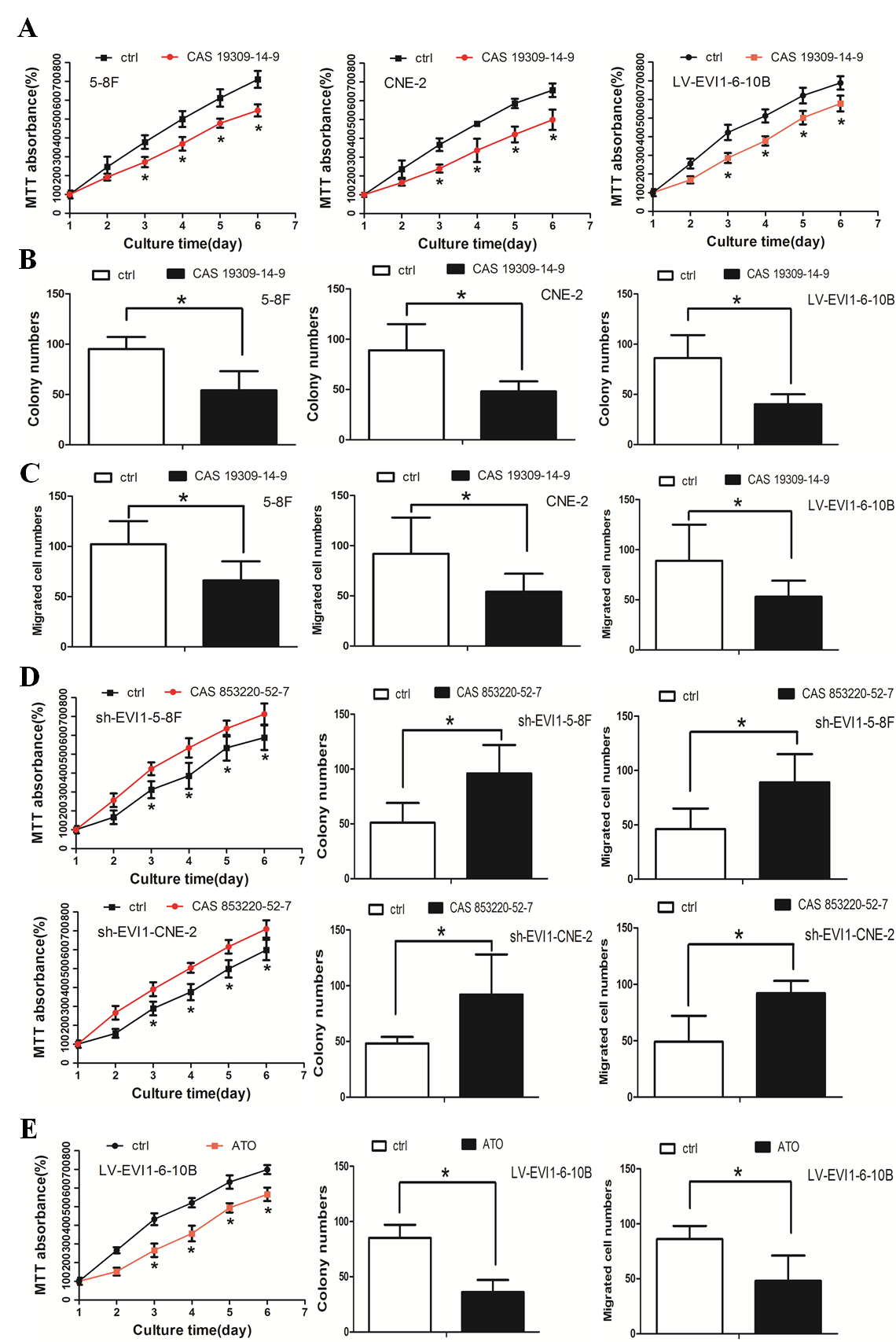

Supplement: Supplementary file 3 — Figure S4. (A) WNT inhibitor drug Cardamonin (CAS 19309–14-9) decreased cell proliferation in 5-8F, CNE-2 and LV-EVI1–6-10B cells, as revealed by MTT assay. (B) WNT inhibitor drug Cardamonin (CAS 19309–14-9) impaired colony formation ability of 5-8F, CNE-2 and LV-EVI1–6-10B cells. (C) The transwell assay revealed that WNT inhibitor drug Cardamonin (CAS 19309–14-9) decreased cell invasion ability of 5-8F, CNE-2 and LV-EVI1–6-10B cells. (D) Wnt agonist drug CAS 853220–52-7 reinforced cell growth, colony formation and invasion ability in sh-EVI1–5-8F and sh-EVI1-CNE-2 cells. (E) EVI-1 overexpression effect on cell growth, colony formation and invasion ability could be partly counteracted by ATO treatment. (TIF 6304 kb) [file 13046_2019_1077_MOESM3_ESM.tif]

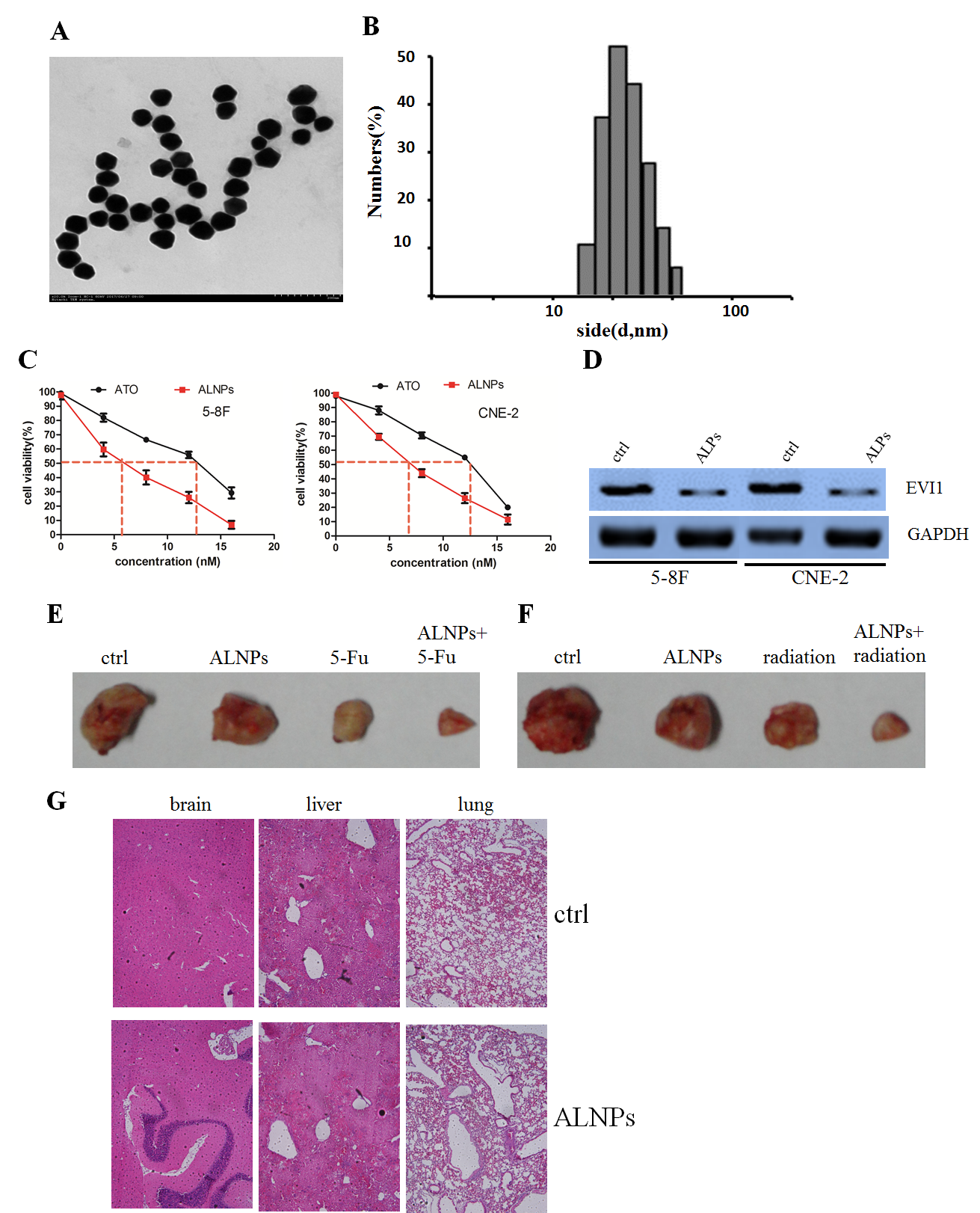

Supplement: Supplementary file 4 — Figure S3. (A) TEM images revealed that the ALNPs were uniform in size distribution with core-shell nanostructures. (B) The size of ALNPs was approximately 50–60 nm as determined by DLS. (C) Compared with free ATO, the ALNP drug delivery system significantly elevated the cytotoxicity to NPC cells as revealed by an MTT assay. (D) ALNPs degraded the EVI1 protein in NPC cell lines. (E)-(F) ALNPs have synergistic effects with both 5-Fu and radiation. (G) H&E staining of tissue sections from the main organs of mice in the PBS- and ALNP-treated groups. (TIF 7703 kb) [file 13046_2019_1077_MOESM4_ESM.tif]
